# Adeno-associated virus-mediated doxycycline-regulatable TRAIL expression suppresses growth of human breast carcinoma in nude mice

**DOI:** 10.1186/1471-2407-12-153

**Published:** 2012-04-24

**Authors:** Liu Zheng, Zhang Weilun, Jiang Minghong, Zhang Yaxi, Liu Shilian, Liu Yanxin, Zheng Dexian

**Affiliations:** 1 National Laboratory of Medical Molecular Biology, Institute of Basic Medical Sciences, Chinese Academy of Medical Sciences & Peking Union Medical College, 5 Dong Dan San Tiao, Beijing, 100005, China

**Keywords:** Controllable gene expression, Tet-On, TRAIL, Adeno-associated virus, Gene therapy

## Abstract

**Background:**

Tumor necrosis factor-related apoptosis-inducing ligand (TRAIL) functions as a cytokine to selectively kill various cancer cells without toxicity to most normal cells. Numerous studies have demonstrated the potential use of recombinant soluble TRAIL as a cancer therapeutic agent. We have showed previous administration of a recombinant adeno-associated virus (rAAV) vector expressing soluble TRAIL results in an efficient suppression of human tumor growth in nude mice. In the present study, we introduced Tet-On gene expression system into the rAAV vector to control the soluble TRAIL expression and evaluate the efficiency of the system in cancer gene therapy.

**Methods:**

Controllability of the Tet-On system was determined by luciferase activity assay, and Western blotting and enzyme-linked immunoabsorbent assay. Cell viability was determined by MTT assay. The breast cancer xenograft animal model was established and recombinant virus was administrated through tail vein injection to evaluate the tumoricidal activity.

**Results:**

The expression of soluble TRAIL could be strictly controlled by the Tet-On system in both normal and cancer cells. Transduction of human cancer cell lines with rAAV-TRE-TRAIL&rAAV-Tet-On under the presence of inducer doxycycline resulted in a considerable cell death by apoptosis. Intravenous injection of the recombinant virus efficiently suppressed the growth of human breast carcinoma in nude mice when activated by doxycycline.

**Conclusion:**

These data suggest that rAAV-mediated soluble TRAIL expression under the control of the Tet-On system is a promising strategy for breast cancer therapy.

## Background

Tumor necrosis factor-related apoptosis-inducing ligand (TRAIL) functions as a cytokine to selectively kill various cancer cells without toxicity to most normal cells [1,2]. Numerous studies have demonstrated the potential use of TRAIL as a cancer therapeutic and a number of TRAIL gene therapy strategies using recombinant viral vectors are described in the literature [3-19]. Our previous study showed that administration of the recombinant adeno-associated virus (rAAV) vector expressing soluble TRAIL (sTRAIL) results in efficient suppression of tumor growth transplanted in the liver and lung of mouse models [16-19].

However, the potential utility and safety of TRAIL has been questioned because of its potential toxicity to normal cells and the mechanism of toxicity still hangs in doubt. Several reports showed that recombinant soluble TRAIL was toxic to normal liver cells [20], keratinocytes[21], brain cells[22], prostate epithelial cells[23], as well as neutrophils[24]. Furthermore, the physiological function of TRAIL is much less well understood. It was reported that TRAIL plays a critical role in innate immunity, adaptive immunity, infectious diseases and autoimmune diseases [25,26], suggesting that TRAIL serves as an important immune regulatory factor under physiological condition. Therefore, systemic over-expression of TRAIL is likely to interfere with normal immune functions. We have shown previously that TRAIL can induce secretion of inflammatory chemokines both in vitro and in vivo [27,28]. In addition, a study indicated that increased hepatic TRAIL expression was accompanied by an increase in liver accumulation of natural killer cells and natural killer T lymphocytes, therefore, mediated liver injury by the innate immune system in certain pathological conditions [29]. One of the key points in successfully implementing gene therapy or biological therapy in the clinical setting is to be able to regulate the targeted gene expression strictly and consistently as and when it is needed.

The Tet-On regulable gene expression system has been widely known to optimize gene therapy strategies [30]. This system can switch targeted gene expression on and off in the presence or absence of the inducer doxycycline (Dox). In the on situation, upon delivery or presence of Dox, reverse tetracycline transactivator (rtTA) activates tetracycline responsive element (TRE) and induces targeted gene expression. In the off situation, upon removal or absence of Dox, rtTA impedes transcript initiation by the TRE promoter.

In the present study we introduced the Tet-On system into the rAAV vector (AAV-TRE-TRAIL&AAV-Tet-On) to control the soluble TRAIL expression and evaluate its efficiency in breast cancer gene therapy. Our data demonstrated that the expression of sTRAIL could be strictly controlled by the system in both normal and cancer cells. As a result the expression could be shut down when side effects appear so as to avoid serious adverse events. Moreover, the gene therapy suppressed breast cancer growth in nude mice efficiently, suggesting that AAV-mediated, Dox regulatable sTRAIL expression is a promising strategy for cancer gene therapy.

## Methods

### Cell lines

MDA-MB-231 (human breast carcinoma) and A549 (human lung carcinoma) cell lines were purchased from American Type Culture Collection (Rockville, MD). HepG2 (hepatocellular carcinoma) was purchased from the Cell Culture Center, Chinese Academy of Medical Sciences (Beijing, China). Human hepatocellular carcinoma SMMC-7721 and SMMC-7402 cell lines were purchased from the Institute of Cell and Biochemistry, Chinese Academy of Sciences (Shanghai, China). These cell lines were cultured in RPMI1640 medium (Gibco, BRL). Human embryo kidney cell line HEK293T/17 was originally purchased from ATCC (No. CRL 1573) and maintained in Dulbecco’s Modified Eagle’s medium (DMEM, Gibco, BRL). U251 (human glioma) was obtained from the Cell Culture Center, Chinese Academy of Medical Sciences (Beijing, China) and maintained in minimal essential medium (MEM) containing Earle’s balanced salt solution (MEM-EBSS, Gibco, BRL). These cells were cultured in the medium supplemented with 10% FBS (Hyclone Laboratories), 100 units/mL of penicillin, 100 μg/mL of streptomycin, and 2 mmol/L of glutamine at 37°C in a 5% CO_2_ incubator.

### Generation of rAAV viral particles

Adeno-associated virus (AAV) vector, a safe vector with fewer pathogenic effects that mediate a long-term targeted gene expression, was used in this study. Recombinant AAV (rAAV) plasmids were constructed using the AAV2 expression vector (a gift from the University of Hong Kong, Hong Kong) containing CAG (chicken β-actin promoter plus cytomegalovirus enhancer) promoter as the backbone. The TRE and CMV-rtTA fragments were cut out from pTRE-tight and pTet-On (Clontech, CA), respectively, and inserted into AAV2 to replace the CAG protmoter. The cDNA encoding soluble TRAIL was amplified by PCR and inserted into AAV2 expression vector under the control of TRE promoter. The resulting constructs were designated as rAAV-TRE-TRAIL&rAAV-Tet-On. The plasmid rAAV-TRE-EGFP encoding enhanced green fluorescent protein (EGFP) and rAAV-TRE-Luc encoding luciferase were constructed as controls. The constructs were validated by DNA sequencing analysis. Packaging and purification of the rAAV particles were performed as described previously[17]. Infectious AAV particles were generated in human embryo kidney cells at a temperature of 37°C. Titers of the rAAV particles were represented as genome particles (Gps)/ml.

### rAAV infection and induction of targeted gene expression

Cells were cultured at 1 × 10^4^ cells per well in 96 well plates or 2 × 10^5^ cells per well in six well plates. The cells were infected with rAAV particles for at least six hours in serum-free and antibiotics-free medium. The ratio of viral particles and cells was 2 × 10^5^ Gps per cell. The infected cells were incubated with or without 1 μg/ml doxycycline (Dox) in complete medium for 24 h, 48 h, 72 h or 96 h, respectively. The relative light units of luciferase gene expression were determined by Modulus Microplate Multimode Reader. The GFP expression was observed under a fluorescence microscope (Nikon TE2000, JP). TRAIL expression was detected by Western blot assay and ELISA with TRAIL/TNFSF10 immunoassay kit (Quantikine, R&D) according to the manufacturer instructions.

### Cell viability assay

Cell viability was evaluated by MTT (3-(4,5-dimethylthiazol-2-yl)-2, 5-diphenyl tetrazolium bromide) (Sigma, St. Louis, MO) assay. MTT assay was followed to manufacturer’s protocol and performed as described previously[16].

### Apoptosis assay

Apoptosis was detected with Annexin V-FITC/PI apoptosis detection kit (BD Biosciences, San Jose, CA) according to the manufacturer’s instructions. Cells were seeded in six-well plates at 2 × 10^5^ cells per well. The cells infected with rAAV-TRE-TRAIL&rAAV-Tet-On were harvested 48 h post infection. Annexin V-FITC/PI staining was performed as described previously[16].

### RT-PCR and western blot analysis

Total RNA were extracted from the cells infected with rAAV-TRE-TRAIL&rAAV-Tet-On, rAAV-TRE-EGFP&rAAV-Tet-On or rAAV-CAG-EGFP particles by using TRIZOL reagent (Invitrogen, Carlsbad, CA) according to the manufacturer’s instructions. The transcriptional expression of WPRE, a characteristic sequence of rAAV vector, regulated by TRE promoter in the cells was analyzed by RT-PCR representative of the targeted gene TRAIL expression. Two micrograms of total RNAs were used to synthesize cDNA in 20 μl reaction mixture with oligo-(dT) as primer and M-MuLV reverse transcriptase followed to manufacturer’s protocol (New England Biolabs, Beverly, MA). Two micrograms of RT product and WPRE-specific primers (5′-AAGATTGACTGGTATTCT-3′ and 5′-ATCCGACTCGTCTGAG-3′) were used in 20 μl reaction mixtures for PCR to detect the transcriptional expression of TRAIL. β-actin was used as an internal control. Western blot analyses were performed as described previously[16]. Rabbit polyclonal antibodies against human TRAIL were pruchased from Santa Cruz Biotechnology (sc-7877; Santa Cruz, CA).

### Animal studies

All animal procedures were approved by the Committee on the use and care of animals, Chinese Academy of Medical Sciences (Beijing). Four week’s old female BALB/c nude mice were recruited for the animal studies. To evaluate the tumoricidal activity of rAAV-TRE-TRAIL&rAAV-Tet-On, rAAV-CAG-TRAIL or rAAV-TRE-EGFP&rAAV-Tet-On, a breast cancer xenograft animal model was established by injecting 1 × 10^7^ MDA-MB-231 cells into mouse dorsal flanks. When the tumor size reached about 50 mm^3^, the animals were divided into three groups (n = 6) and intra-tail vein injections of 4 × 10^11^ Gps of rAAV-TRE-TRAIL&rAAV-Tet-On (TRE-TRAIL group), rAAV-CAG-TRAIL (CAG-TRAIL group) or rAAV-TRE-EGFP&rAAV-Tet-On (TRE-EGFP group) particles were administered. The ratio of rAAV-TRE and rAAV-Tet-On particles in TRE-TRAIL and TRE-EGFP group was 1:1. Dox was added into the mouse drinking water at a concentration of 1 mg/ml after injection. Tumor growth was observed every 4 days for 32 days by measuring the two dimensional longest axis (a) and shortest axis (b) with a caliper when the tumor was macroscopic. The tumor volume was calculated by using the following formula: volume in mm^3^ = (ab^2^)/2. At the end of experiment, the animals were sacrificed, tumors and tissues were removed for RT-PCR and Western blot analysis.

### Statistics

Results of the data are reported as the mean ± standard deviation (SD) and ANOVA for repeated data was used to analyze the differences between the groups using statistical software SPSS10.0. The significant level was defined as *p* < 0.05.

## Results

### Controllable expression of target gene driven by tet-on system

**Figure 1:**
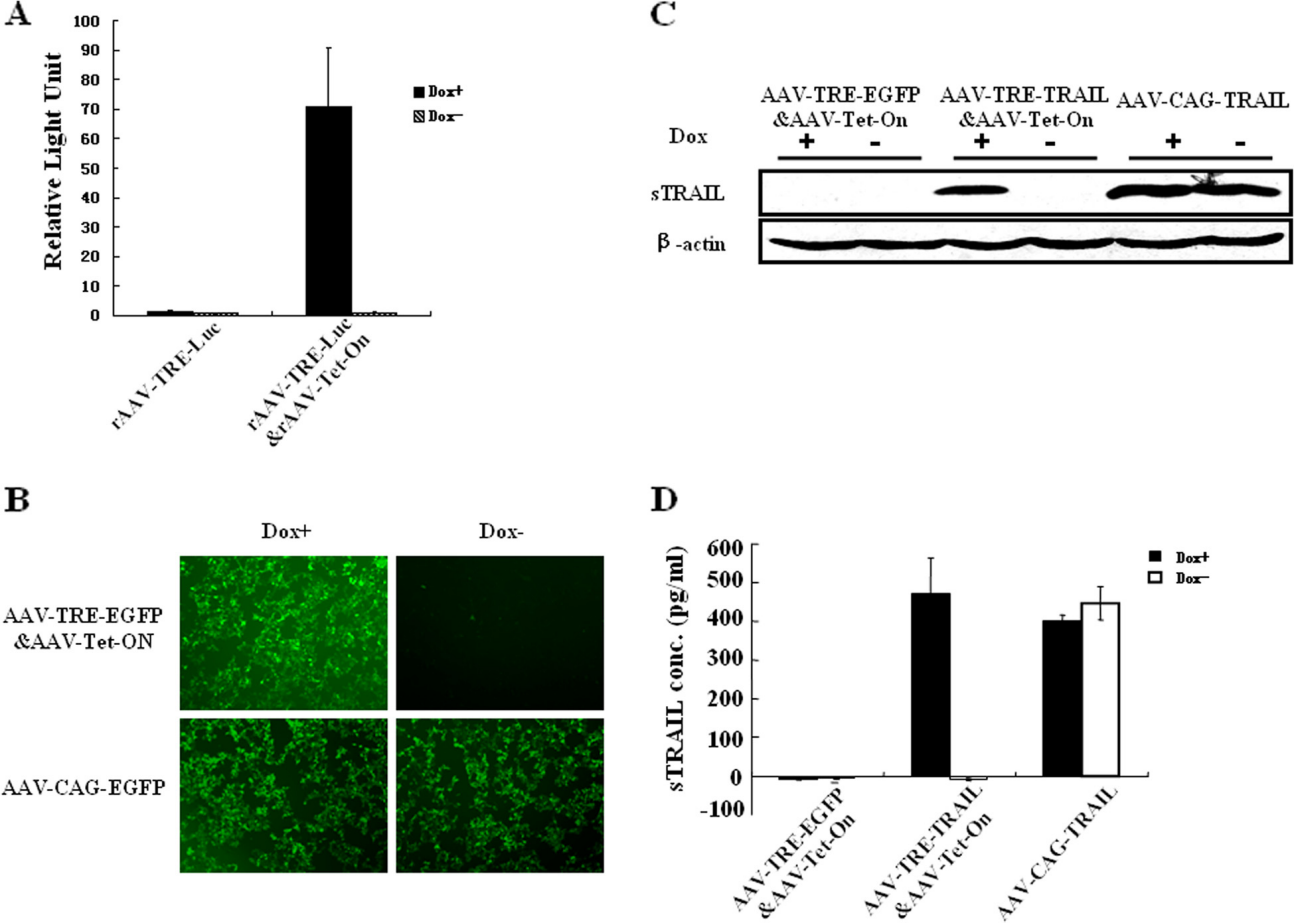
**AAV-TRE&AAV-Tet-On system regulates targeted gene expression.** HEK293T/17 cells were infected with rAAV-TRE-Luc&AAV-Tet-On or rAAV-TRE-Luc for 48 h, respectively. rAAV-CAG-EGFP were used as positive control. (**A**) The relative light units (RLU) representing luciferase expression in the cells infected with rAAV-TRE-Luc&AAV-Tet-On/Dox^+^ were detemined on Modulus Microplate Multimode Reader. (**B**) The EGFP expression in HEK293T/17 cells infected with rAAV-TRE-EGFP&AAV-Tet-On was observed by fluorescence microscopy (100 × magnification). (**C**) TRAIL protein expression in HEK293T/17 cells infected with rAAV-TRE-TRAIL&AAV-Tet-On for 48 h was detected by Western blot assay. β-actin was used as loading control. (**D**) Soluble TRAIL secreted into the medium of HEK293T/17 cells at 96 h after the transduction was determined by ELISA. The cells infected with rAAV-TRE-EGFP&AAV-Tet-On were used as negative control. The cells infected with rAAV-CAG-TRAIL were used as positive control. Dox+, 1 μg/ml doxycycline in cell culture medium. Dox-, without doxycycline in the medium.

To evaluate a controllable gene expression of the target gene driven by the Tet-On system, HEK293T/17 cells were first transduced with the recombinant virus rAAV-TRE-Luc&AAV-Tet-On at 2 × 10^5^ Gps per cell.The relative light units representing luciferase activity in the cell lysate were detected. As shown in Figure 1A, the luciferase activity in the cells induced with 1 μg/ml of doxcycline (AAV-TRE-Luc&AAV-Tet-On/Dox^+^) was 70 fold higher than the cells without Dox treatment (AAV-TRE-Luc&AAV-Tet-On/ Dox^-^). However, there was no difference in the cells transduced with rAAV-TRE-Luc only under the presence (Dox^+^) or absence (Dox^-^) of Dox. This result was confirmed in the cells transduced with AAV-TRE-EGFP&AAV-Tet-On (Figure 1B), in which GFP expression driven by TRE and observed by fluorescence microscopy was as strong as that driven by CAG in AAV-CAG-EGFP [17]. These data indicate that Tet-On system can strictly control target gene expression driven by TRE promoter.

Then, we examined the control capability and intensity of the rAAV-mediated soluble TRAIL expression driven by TRE promoter in the AAV-TRE-TRAIL&rAAV-Tet-On recombinant virus. TRAIL expression in the cells was detected by Western blot and soluble TRAIL secreted in the media by ELISA. AAV-TRE-EGFP&AAV-Tet-On and AAV-CAG-TRAIL were used as negative control and positive control, respectively. As shown in Figure 1C, the expression of sTRAIL was clearly observed in HEK 293 T/17 cells transduced with rAAV-TRE-TRAIL&AAV-Tet-On and induced by Dox (Dox^+^), but not in those cells without Dox induction (Dox^-^). The sTRAIL in the culture media of AAV-TRE-TRAIL&AAV-Tet-On/Dox^+^ was 469.9 pg/ml, while there was no detectable sTRAIL in the media of AAV-TRE-TRAIL&AAV-Tet-On/Dox^-^ (Figure 1D). These data suggest that AAV-TRE-TRAIL&AAV-Tet-On system effectively controls sTRAIL gene expression.

### Tet-on system-controlled sTRAIL expression suppresses tumor cell growth in vitro

**Figure 2:**
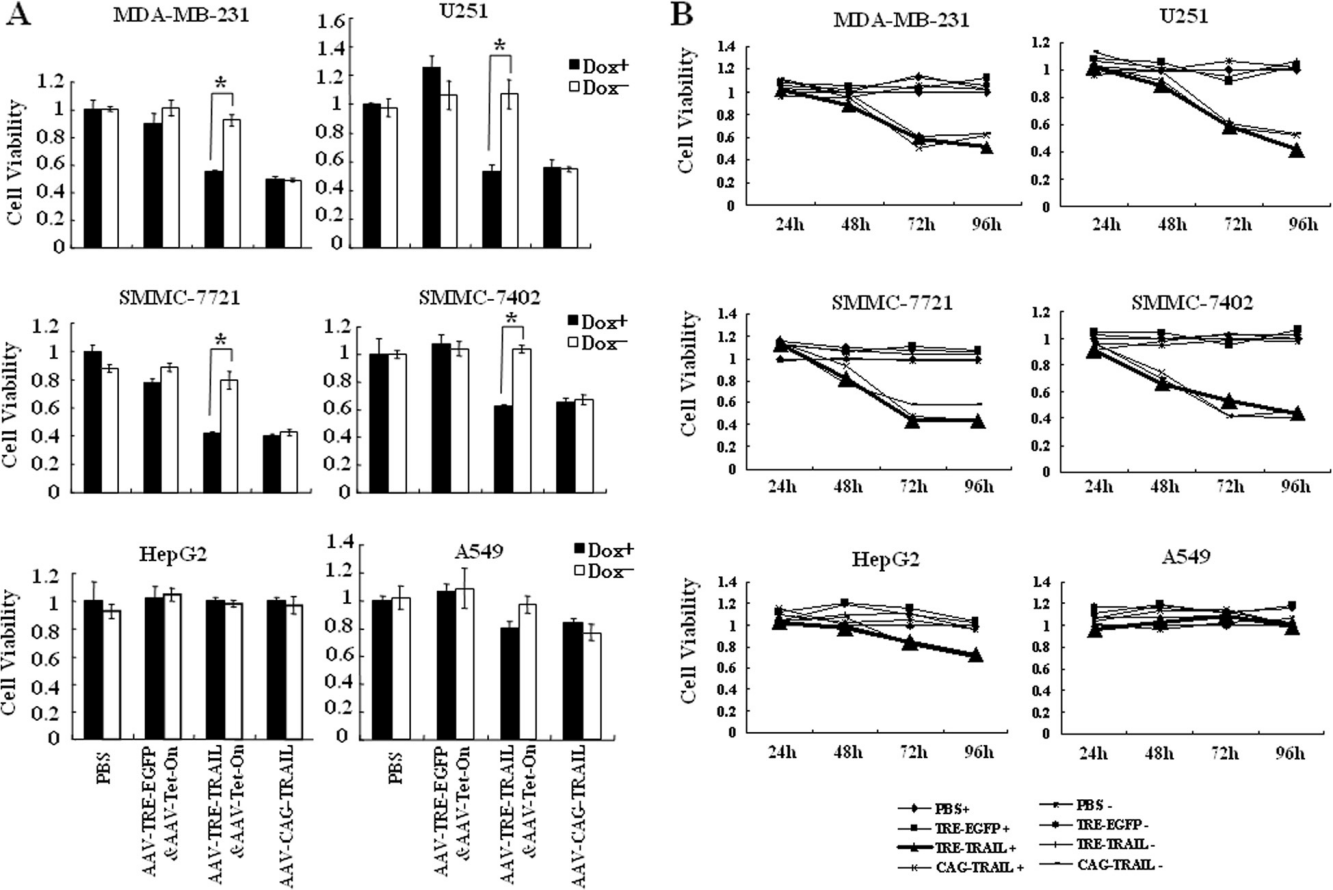
**Tet-On system-controlled sTRAIL expression induces apoptosis in tumor cells.** (**A**) MDA-MB-231, U251, SMMC-7721, SMMC-7402, A549 and HepG2 cells were exposed for 24 h to the supernatant of HEK293T cells that have infected with rAAV-TRE-TRAIL&AAV-Tet-On at 2 × 105 GPs per cell for 72 h. Supernatant of HEK293T cells infected with rAAV-TRE- EGFP&AAV-Tet-On and rAAV-CAG-TRAIL were used as negative and positive control, respectively. Cell viability was measured by MTT assay. Results are presented as the mean ± standard deviation of three individual experiments in each group. Dox+, 1 μg/ml doxycycline in the cell culture medium; Dox-, without doxycycline in the medium. (**B**) Infection with rAAV-TRE-TRAIL&AAV-Tet-On/Dox + induced apoptosis and the time course of virus infection.

To evaluate the ability of the Tet-On system-controlled sTRAIL expression to suppress tumor cell growth, tumor cell lines of MDA-MB-231, U251, SMMC-7721, SMMC-7402, A549 and HepG2 were exposed for 24 h to the supernatant of HEK293T cells that have been infected with rAAV-TRE-TRAIL&AAV-Tet-On particles at 2 × 10^5^ GPs per cell for 72 h. Supernatant of HEK293T cells infected with rAAV-TRE- EGFP&AAV-Tet-On and rAAV-CAG-TRAIL were used as negative and positive control, respectively. The viability of tumor cells was evaluated by MTT assay. As shown in Figure 2A, the viability of MDA-MB-231, U251, SMMC-7721 and SMMC-7402 cells infected with rAAV-TRE-TRAIL&AAV-Tet-On in the presence of Dox decreased to 55% ± 1.4%, 52.9% ± 5.1%, 41.6% ± 6.4% and 62.1% ± 1.4%, respectively. In contrast, the viability of the cells infected with rAAV-TRE-TRAIL&AAV-Tet-On in the absence of Dox was no change compared with the negative control. We further demonstrated the viability decrease in MDA-MB-231, U251, SMMC-7721 and SMMC-7402 cells directly infected with rAAV-TRE-TRAIL&AAV-Tet-On/Dox^+^ was correlated with the viral infection (Figure 2B).

**Figure 3:**
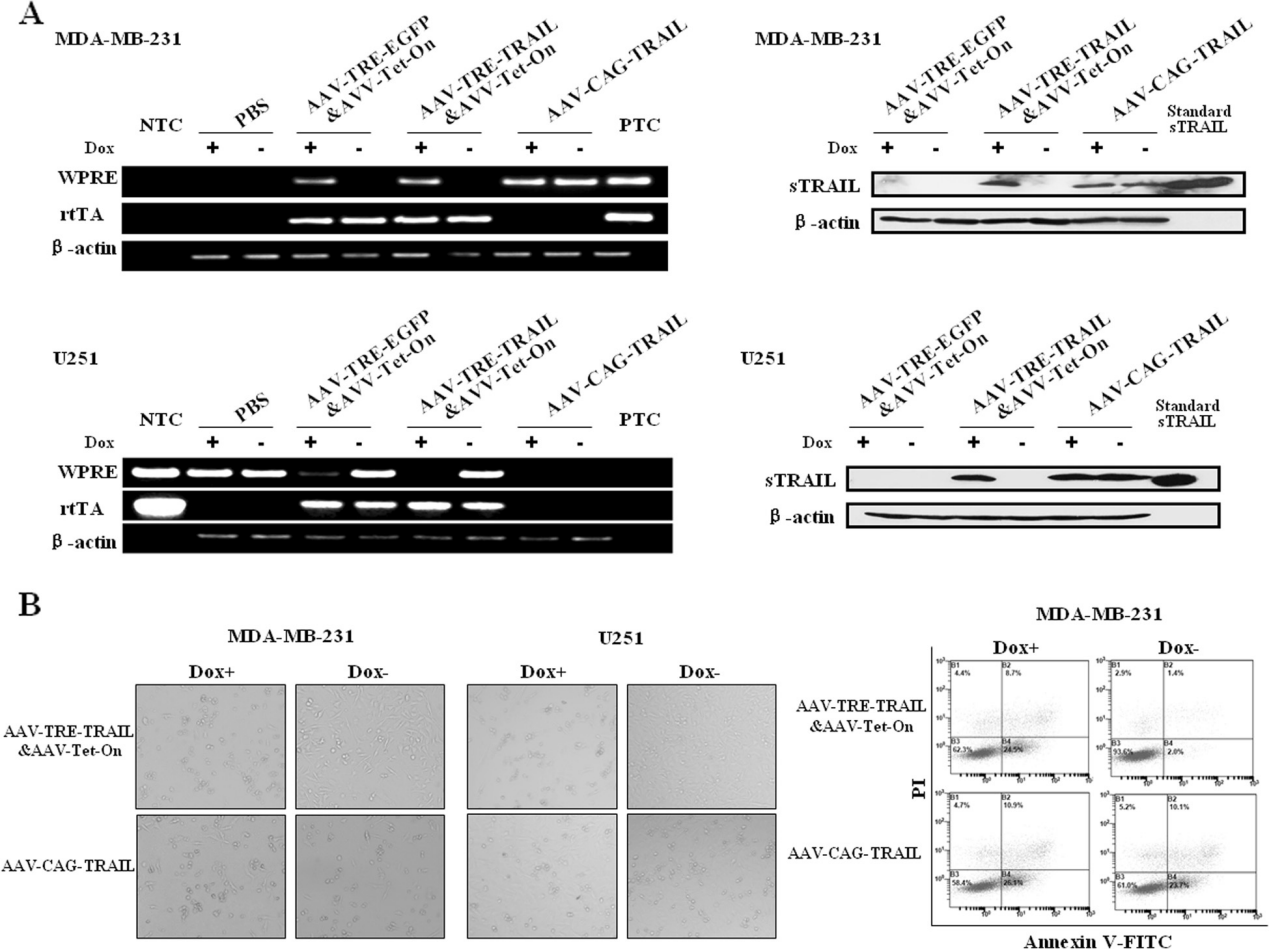
**Tet-On system-controlled sTRAIL expression suppresses tumor cell growth by apoptosis.** MDA-MB-231 and U251 cells were transduced for 48 h with rAAV-TRE-TRAIL&AAV-Tet-On at 2 × 10^5^ GPs per cell. rAAV-TRE- EGFP&AAV-Tet-On and rAAV-CAG-TRAIL were used as negative and positive control, respectively. (**A**) The sTRAIL expression was detected by RT-PCR and Western blot assay. (**B**) The viability of tumor cells was evaluated by microscopy and flow cytometry. Dox+, 1 μg/ml doxycycline in the medium. Dox-, without doxycycline in the medium. WPRE, woodchuck hepatitis virus posttranscriptional regulatory element, represents sTRAIL mRNA expression. NTC: No-Template Control. PTC: Positive Template Control.

Apoptosis assay by Annexin V-FITC/PI double staining followed by microscopy and flow cytometry showed that while Dox induced-TRAIL expression was detected by both RT-PCR and Western blot assay in U251 and MDA-MB-231 cells (Figure 3A), tumor cell death by apoptosis was occurred markedly (Figure 3B). These data suggest that sTRAIL expression mediated by rAAV-TRE-TRAIL&rAAV-Tet-On induces tumor cell death by apoptosis, which is strictly regulated by Tet-On system.

### Tet-on system-controlled sTRAIL expression efficiently suppresses human tumor growth in vivo

**Figure 4:**
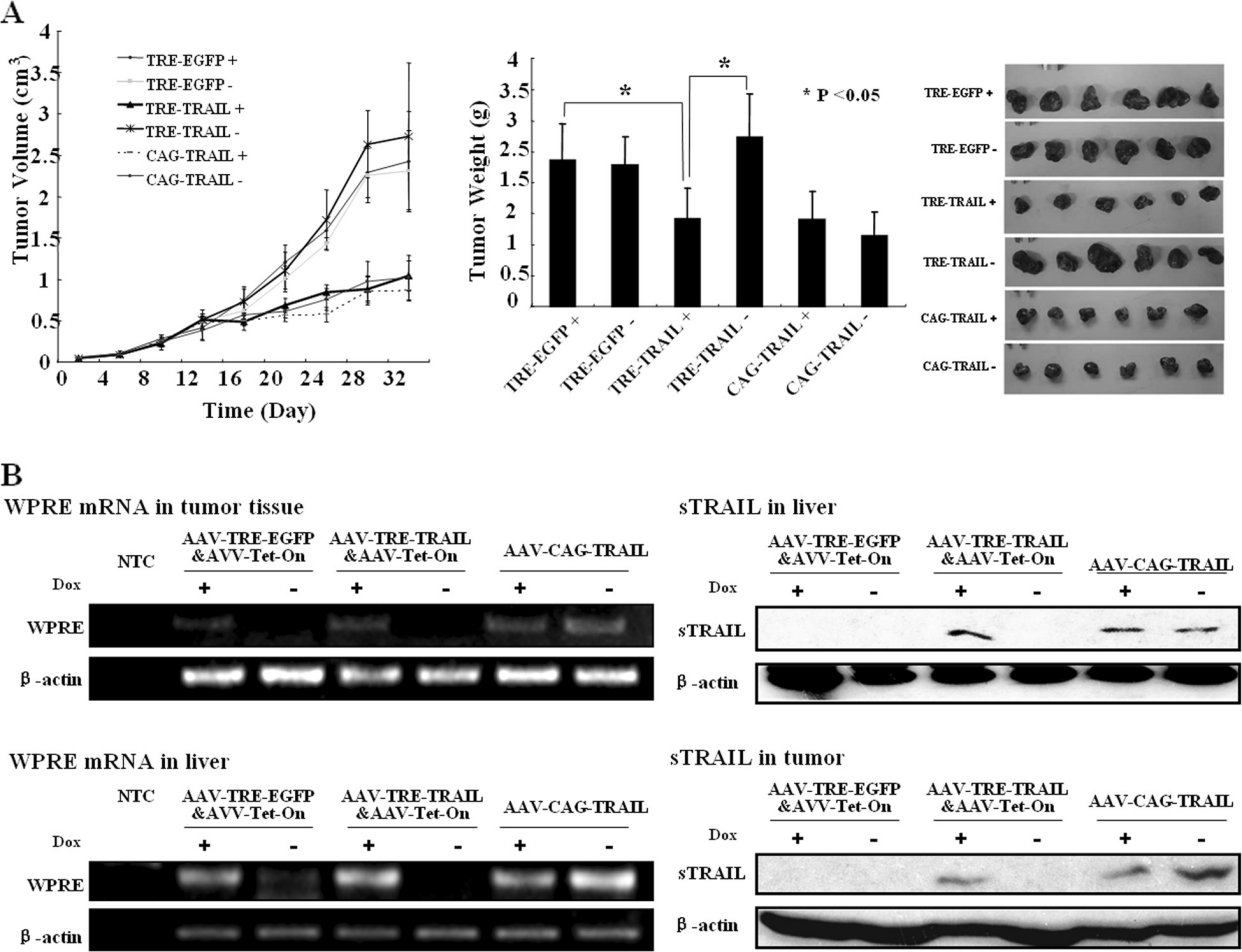
**Tet-On system-controlled sTRAIL expression suppresses the growth of MDA-MB-231 xenografts in nude mice.** A xenograft MDA-MB-231 animal model was established in nude mice by subcutaneous transplantation. When the tumor size reached to about 50 mm^3^ (about 7 days post-inoculation), the animals were divided into six groups (n = 6). The rAAV-TRE-TRAIL&rAAV-Tet-On particles of 4 × 10^11^ Gps were administered through tail vein injection and the animals (day 0) were fed with drinking water with or without doxycycline (1 mg/ml). (**A**) The tumor growth was observed by measuring the tumor size every four days for total 32 days. The rAAV-TRE-EGFP&rAAV-Tet-On and rAAV-CAG-TRAIL particles were administered as negative and positive control, respectively. The animals were sacrificed and the tumor weights (**A**) were measured 32 days after virus administration (day 32). Results are presented as the mean ± standard deviation of 6 mice in each group. *P <0.05. (**B**) The sTRAIL expression in tumor tissue was examined by reverse transcriptional PCR and Western blot assay. β-actin served as protein loading control. WPRE, woodchuck hepatitis virus posttranscriptional regulatory element, representing sTRAIL mRNA expression. NTC: No-Template Control. PTC: Positive Template Control.

To test the tumor suppressing efficacy of rAAV-TRE-TRAIL&rAAV-Tet-On in vivo, we established a xenograft animal model in nude mice by subcutaneous transplantation of MDA-MB-231 breast cancer cells, which was relatively sensitive to the viral infection. When the tumor size reached about 50 mm^3^, the animals were divided into six groups (Table 1, n = 6). The rAAV-TRE-TRAIL&rAAV-Tet-On particles of 4 × 10^11^ Gps were administered through tail vein injection and the animals were given drinking water with or without Dox. The tumor growth was observed by measuring the tumor size every four days for a total of 32 days. The rAAV-TRE-EGFP&rAAV-Tet-On and rAAV-CAG-TRAIL particles were administrated as negative and positive control, respectively. As shown in Figure 4A, tumor volumes and weights in the Dox^+^ group were smaller than that in the Dox^-^ group (*p* < 0.05). Furthermore, the therapeutic efficacy in Dox^+^ group was similar to the rAAV-CAG-TRAIL positive control. Western blot analysis showed the presence of sTRAIL protein in tumor tissue and liver in the animals of Dox^+^ group, but not in those of Dox^-^ group (Figure 4B). These data indicate that Dox-induced sTRAIL expression suppresses subcutaneous breast cancer growth significantly.

**Table 1:** Animal groups and administration

Animal Group	Administration	Dox*	n
**TRE-EGFP+**	AAV-TRE-EGFP&AAV-Tet-On	+	6
**TRE-EGFP -**	AAV-TRE-EGFP &AAV-Tet-On	–	6
**TRE-TRAIL+**	AAV-TRE-TRAIL&AAV-Tet-On	+	6
**TRE-TRAIL-**	AAV-TRE-TRAIL&AAV-Tet-On	–	6
**CAG-TRAIL+**	AAV-CAG-TRAIL	+	6
**CAG-TRAIL-**	AAV-CAG-TRAIL	-	6

* Dox+, 1 mg/ml in drinking water; Dox^-^, without Dox in drinking water.

### Tet-on system-controlled sTRAIL expression induces tumor cell death in vivo by apoptosis via classic apoptosis pathway

**Figure 5:**
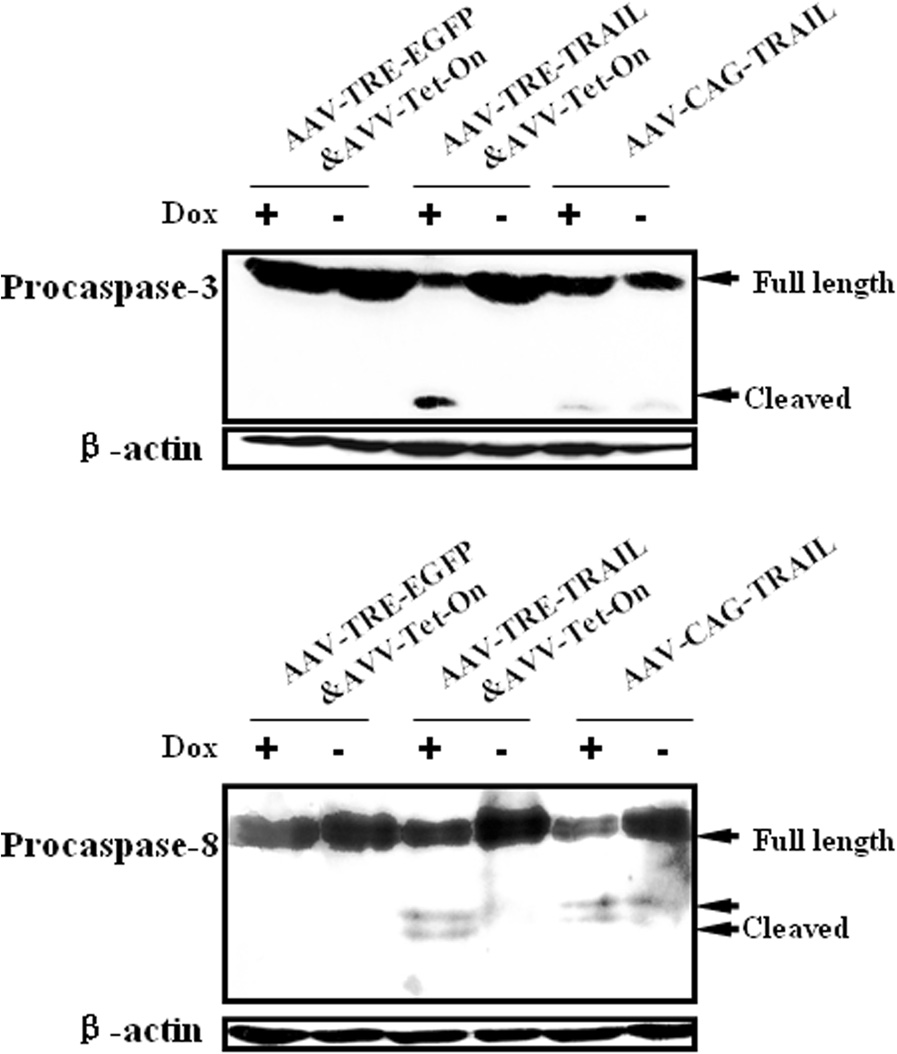
**Tet-On system-controlled sTRAIL expression induces tumor cell apoptosis in vivo.** The xenograft tumor cells were isolated from the animals administered with rAAV-TRE-TRAIL&rAAV-Tet-On and lyzed with lysis buffer. 400 micrograms of total protein in the lysed were subjected to SDS-PAGE. Cleavage of procaspase-8 and procaspase-3 were examined by Western blot assay using corresponding specific antibodies. β-actin was used as loading control. The tumor lysates from the animals administrated with rAAV-TRE-EGFP&rAAV-Tet-On and rAAV-CAG-TRAIL were used as negative and positive control, respectively.

To explore the mechanism of tumor growth suppression mediated by rAAV-TRE- TRAIL&rAAV-Tet-On infection, cleavage of pro-caspases, a hallmark of cell death by apoptosis, in xenograft tumor tissue from the animals was further detected by Western blot analysis. As shown in Figure 5, rAAV-TRE-TRAIL&rAAV-Tet-On administration and Dox induction resulted in the cleavage of procaspase-3 and procaspase-8, but not in the animals without Dox in the water, indicating that Tet-On system-controlled sTRAIL expression in the animals induces tumor cell death by apoptosis via the classic apoptosis pathway characterized by caspase activation.

## Discussion

Tumor necrosis factor-related apoptosis-inducing ligand (TRAIL) is a target for cancer therapy because of its ability to induce apoptosis in malignant cells while not in most normal cells and tissues. However, its potential toxicity on some normal human cells (e.g. hepatocytes) has been questioned, but the mechanism of the related toxicity still hangs in doubt. Furthermore, the physiological function of TRAIL is much less well understood. From a clinical point of view, one of the key issues in successfully implementing cancer gene therapies or biological therapies in the clinical setting is to be able to regulate gene expression strictly and consistently as and when it is needed.

In the present study, we introduced the Tet-On regulatable gene expression system into rAAV vector (AAV-TRE-TRAIL&AAV-Tet-On) to control the soluble TRAIL expression and evaluate the efficacy in cancer gene therapy. We observed that the expression and secretion of sTRAIL could be strictly controlled by the Tet-On system in both normal and cancer cells. Transduction of various cancer cell lines with AAV-TRE-TRAIL&AAV-Tet-On under the presence of Dox resulted in significant cell death by apoptosis in SMMC-7721, SMMC-7402 liver cancer cells, and A549 lung cancer cells, but not in HepG2 liver cancer cells (Figure 2A). The resistance of HepG2 to AAV-TRE-TRAIL&AAV-Tet-On might be a result of inefficient of rAAV transduction or intracellular distinguishable events with TRAIL and its receptors in the cells.

Intravenous injection of rAAV in the cancer mouse models showed that sTRAIL protein was predominantly expressed in liver and tumor tissue under induction condition (Figure 4B). The tumor weights and volumes were obviously smaller in the animals fed with Dox compared with those without Dox. As a result, the expression could be shut down when side effects appear so as to avoid serious adverse events.

To confirm whether there is an excellent target gene expression mediated by the AAV vector with the Tet-On gene expression system, we established a positive control of rAAV vectors with a CAG promoter, which has shown a good performance in AAV-mediated gene therapy [17,18]. Data in our research showed the intensity of rAAV-TRE&rAAV-Tet-On mediated target gene expression under the induction condition was similar to that of rAAV-CAG, indicating that the Tet-On gene expression system has considerable capacity in controlling target gene expression.

The Tet-On system offers many advantages over other regulable gene expression systems [30]. The inducer Dox, an analogue of tetracycline, has been used as an antibiotic for decades and it has been well characterized in the clinical setting. It is nontoxic at doses required for gene activation in preclinical and clinical studies, and the margin of safety is high. Dox is metabolized and cleared from the body rapidly, making it an ideal drug for the rapid increase in expression, long-term expression, and rapid decrease in expression of the desired transgene. The components of the Tet-On system recognizes unique fragment of DNA, and Dox does not interfere with native proteins, reducing the potential of serious side effects.

However, a limitation of the approach is that two AAV vectors were used as a mixture. One contained TRAIL regulated by TRE, and the second contained rtTA activated by Dox. If the two AAV do not co-localize equally in tumors, then TRAIL mediated killing would not be optimized. Many researchers have attempted to construct TRE and rtTA into one vector to improve the efficiency of gene transduction. We also tried this approach to ligate rtTA, TRE promoter and TRAIL gene in the rAAV vector, therefore, forms a single rAAV vector, expecting to improve the expression intensity of the transgene. However, this approach challenges the limits of rAAV packaging capacity and causes difficulties in virus packaging. In addition, we found that the cells transduced with the single vector express the transgene in the absence of Dox (data not shown), known as gene expression leakage in regulable gene expression system. Instead, a dual rAAV vector system in which the TRE-TRAIL and rtTA were constructed into two rAAV vectors respectively provided a similar intensity in transgene expression and much more strict controllability compared with the single vector system.

## Conclusions

We provided evidence in this study that the expression of soluble TRAIL could be strictly controlled by the Tet-On system both in vitro and in vivo; the expression could be shut down when side effects appear to avoid potential serious adverse events. Intravenous injection of the recombinant virus efficiently suppressed the growth of human breast carcinoma in nude mice. AAV-mediated sTRAIL expression under the control of Tet-On system is a promising strategy for cancer biological therapy.

## Abbreviations

TRAIL = tumor necrosis factor–related apoptosis-inducing ligand; rAAV = recombinant adeno-associated virus; WPRE = woodchuck hepatitis virus post-transcriptional regulatory element; EGFP = enhanced green fluorescent protein; Dox = doxycycline; MTT = 3-(4, 5-dimethylthiazol-2-yl)-2, 5-diphenyl tetrazolium bromide.

## Misc

Liu Zheng and Zhang Weilun contributed to this work equally.

## Competing interests

The authors declare that they have no competing interests.

## Authors’ contributions

ZL performed the experiments and wrote the manuscript. WLZ carried out the animal experiments and statistical analysis and is co-first author. YXZ participated in the animal experiments and immunoassays. SLL participated in analyzing the data. DXZ and YXL, as the co-corresponding authors, designed the protocol and revised the manuscript. All authors read and approved the final manuscript.

## Pre-publication history

The pre-publication history for this paper can be accessed here:

http://www.biomedcentral.com/1471-2407/12/153/prepub
